# Design and hydrologic performance estimation of highway filter drains using a novel analytical probabilistic model

**DOI:** 10.1038/s41598-024-52760-7

**Published:** 2024-01-29

**Authors:** Aniekan E. Essien, Yiping Guo, Mohamed Khafagy, Sarah E. Dickson‐Anderson

**Affiliations:** 1 Department of Civil Engineering, McMaster University, Hamilton, Ontario L8S4L7 Canada; 2 Irrigation and Hydraulics Department, Faculty of Engineering, Cairo University, Giza, Egypt

**Keywords:** Climate sciences, Environmental sciences, Hydrology, Engineering, Mathematics and computing

## Abstract

Sustainable drainage systems (SuDS) are nature-based methods of managing urban stormwater runoff. Although they are widely used, some SuDS, such as highway filter drains (HFDs), are understudied with respect to sizing and performance. For the first time, we developed an analytical probabilistic model (APM) that can be used to design and estimate the hydrologic performance of HFDs. Unlike the conventionally used design-storm based or continuous simulation approaches, our APM can directly calculate the runoff capture ratios of HFDs using closed-form analytical equations. Validation of the APM presented here shows that it is robust and reliable. The relative differences between the APM-estimated and continuous simulation-determined runoff capture ratios for all the simulated design cases are less than 8.5%.

Globally, rapid and dramatic environmental changes are occurring due to increased urbanization and global warming. Although it can be argued that the increase in urbanization may bring sustainable growth^1^, that can only be achieved if it is properly managed. In addition, one cannot ignore its impacts on stormwater systems. Impervious urban areas tend to generate more and faster runoff from rainfalls, leading to more severe and frequent flooding. To mitigate this, scientists and engineers came up with approaches of what is generally referred to as sustainable urban drainage systems (SuDS) or sustainable drainage systems (SuDS) in the United Kingdom and many parts of Europe, low-impact development (LID) in Northern America and New Zealand, and water sensitive urban design (WSUD) in Australia^2^. Although a unified terminology is desirable, multiple terms exist reflecting localized content^2,3^. In this paper, sustainable drainage systems (SuDS) is the terminology used for this technology.

Filter drain, otherwise known as "French drain", an alternative name for combined surface and sub-surface drain, is one of the most widely used sustainable urban drainage systems (SuDS)^4,5^. One of the core reasons for using this stormwater management system is its potential ability to mimic the natural processes of managing stormwater. The natural processes include attenuation, passive treatment, and filtration. However, unlike infiltration trenches, the primary role of filter drains is not stormwater infiltration^6^. Filter drain is one of the few stormwater management systems, if not the only one, that can be used for all the three control levels, i.e., lot-level, conveyance, and end-of-pipe controls. This unique property of filter drain has kept its relevance to stormwater management in many jurisdictions. In the United Kingdom, filter drains are the principal stormwater management/drainage systems for their major roads. For example, in England, about 4300 miles of motorways (highways) and other major roads, which is arguably the most important and expensive infrastructure of the country^7^, are serviced by filter drains, popularly known as highway filter drains (HFDs)^8^. In fact, it is estimated that HFDs serve 50% of the Strategic Road Network (SRN) in England^3^. In Scotland, 43% of its strategic trunk roads are drained by the same highway drainage system^9^.

The use of filter drains in motorways and other major roads is not only associated with the UK but also with other countries worldwide. For example, this system is well-known as highway edge drains in Canada and the US. In fact, more than 40% of dual carriageways in the Republic of Ireland use HFDs, and Spain has adopted filter drains as an alternative to the traditional road drainage methods^8,10^. This is mainly due to the requirement of removing stormwater from urban roads as soon as possible to avoid flooding and accidents caused by reduced friction between vehicle tires and road surfaces. The same consideration applies to pedestrian walkways, runways, and airport aprons. Water lubrication gets worse in synergy with other pollutants found on major roads, such as microplastics (MPs), Per- and polyfluoroalkyl substances (PFASs), heavy metals, and other hydrocarbons. HFDs are designed to help meet the four stormwater management requirements (i.e., water quality, water quantity, biodiversity, and amenity)^11^. In addition to that, some other advantages of HFDs include^12–15^: (1) low cost and high carbon footprint savings, thereby promoting environmental sustainability; (2) use of local and/or recycled products, thereby improving local economy; (3) most uncomplicated type of highway drainage system to construct; (4) multi-stage and multipurpose drainage system as they can be used during initial road construction of the highway network for surface and subsurface water management; (5) possessing high hydraulic conductivity, thereby provide rapid control and management of stormwater; and (6) possessing increased microbial activities, which help to breakdown trapped pollutants, such as herbicides, oil and grease, etc.

Consequently, the importance of continuously searching for methods to design HFDs to ensure better efficiency and performance, especially when using more straightforward and non-time-consuming tools, can never be over-emphasized. In addition, there is data scarcity on the hydrologic performance of HFDs due to limited research in some of the areas related to the system^8^, and this has raised the question of their proper design and performance. As indicated by Essien et al., using the confirmed exponential distributions of rainfall event characteristics for locations throughout the UK for the sizing and hydrologic performance estimation of HFDs may be possible^16^. This applies to other nations worldwide where the exponential distributions of rainfall event characteristics in some of their locations have been verified, such as Canada, the US, etc^16–20^.

Here, for the first time published, our research is aimed at (1) developing an analytical probabilistic model (APM) for designing HFDs and estimating their hydrologic performances and (2) evaluating the performance of the four band conditions of HFDs established through physical assessment in the UK. Our newly developed APM uses analytical equations to directly determine the performance characteristics of HFDs and is much easier to use than numerical simulation models.

## HFDs design

**Figure 1:**
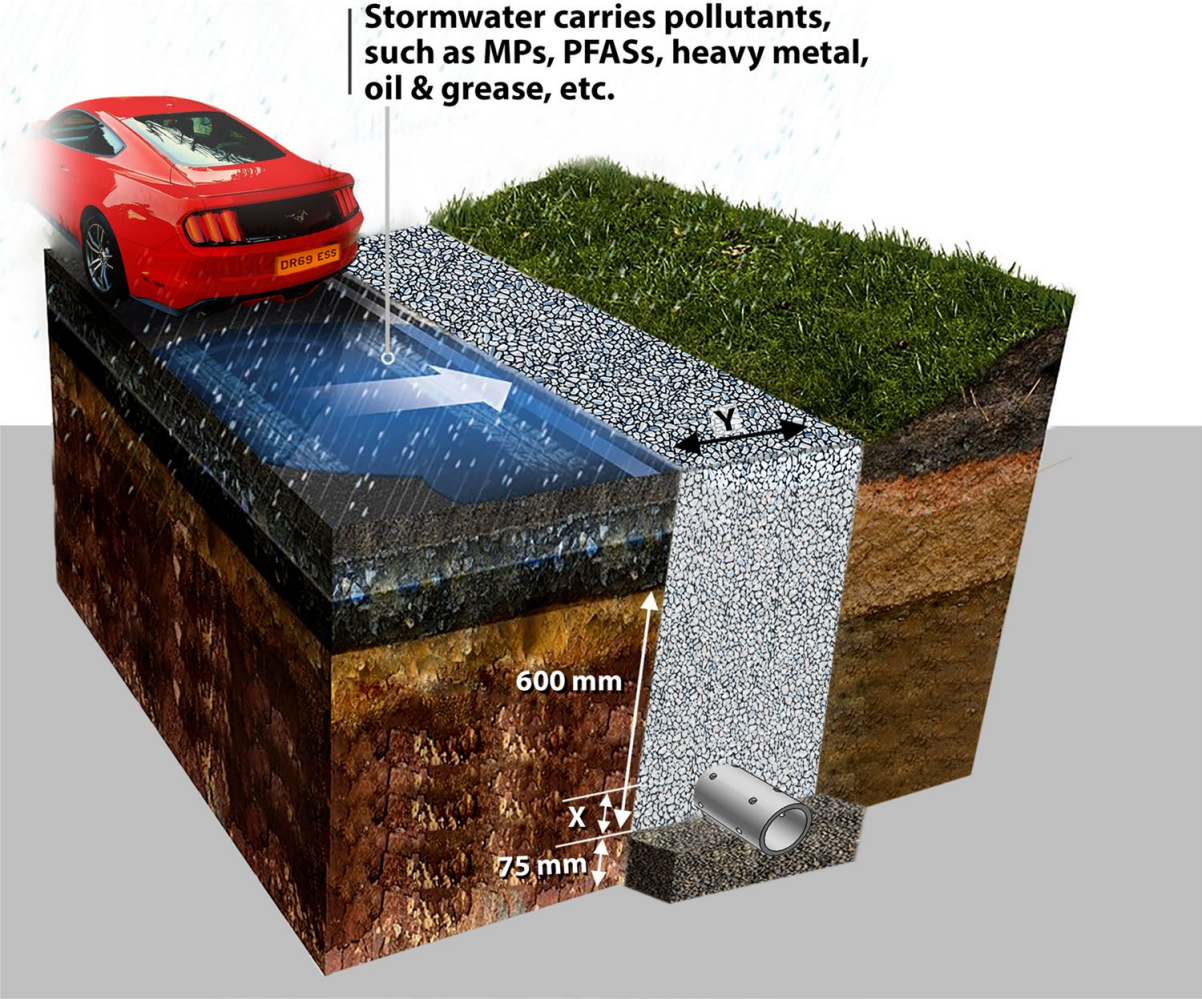
A typical HFD that can be found on motorways in the UK. Y is the width of the HFD, which can be estimated by adding 300 mm to the diameter of the underdrain pipe, and the other dimensions shown in this figure are the minimum requirements for designing HFDs in the UK (Supplementary Section 1). The figure was adapted from an article by SDS Limited published on its website (https://www.sdslimited.com/sds-tackles-highway-metals-pollution/)^32^.

Different jurisdictions have different design requirements for highway drainage systems. Here, we applied our newly developed analytical probabilistic model in a case study using locations within the UK. However, we would like to acknowledge that our model can be used for any region as far as the distribution parameters of the specific location are determined first. The United Kingdom of Great Britain and Northern Ireland is a country of four nations serviced by over 273,500 miles of road network^21,22^. This area was chosen for this research because of the aforementioned extensive use of HFDs on its major roads. In addition, there is currently no published study on designing and estimating the hydrologic performance of HFDs using any similar analytical probabilistic models. Standard HFDs are trenches filled with sorted aggregates fitted with a perforated pipe, that run in parallel with significant parts (verges or central reserves) of motorway networks or other major roads in the UK. Some of the trenches are incorporated with geotextiles and/or enhanced porous media to reduce clogging and improve stormwater treatment. The UK industry standard design criteria for HFDs is 1 m × 1 m for the width and depth^3,6,23^. Figure 1 presents a standard HFD design adopted by Highway England. Other essential design criteria associated with HFDs based on the UK standard requirements and industry practices are reported in the documents referenced here and summarised in Supplementary Section 1^4,6,15,24–31^.

## Derivation of the analytical equations for assessing the hydrologic performance of HFDs

The analytical probabilistic approach starts by analyzing the region's rainfall event characteristics, i.e., event volume ($v$), event duration ($t$), and interevent time ($b$). Actual observed individual rainfall events are obtained by separating extensive continuous historical rainfall records from weather stations at the locations of interest into consecutive events first. To separate continuous rainfall records into individual events, a suitable interevent time definition (IETD) must be selected first. IETD is the minimum time period between rainfall events. Dry periods shorter than the IETD within rainfall episodes are considered to be parts of a single rainfall event, while rainfall episodes separated by a dry period longer than the selected IETD are regarded as different rainfall events. The length of the dry period between individual rainfall events is referred to as the interevent time. Samples of $v, t,\, {\text{and}}\, b$ for a location of interest can be obtained by selecting an appropriate IETD and carrying out the event separation using observed historical rainfall data of that location. For many locations, including some in the UK (which is discussed with the numeric data in the Methods section), the event separations have been done already, and samples of $v, t,\, {\text{and}}\, b$ from different locations have been tested and found that they all fit the following exponential distributions (i.e., Eqs. (1)–(3)):

1 $${f}_{V}\left(v\right)= \zeta {e}^{-\zeta v}, v \ge 0$$

2 $${f}_{T}\left(t\right)=\uplambda {e}^{-{\lambda t}}, t \ge 0$$

3 $${f}_{B}\left(b\right)= \psi {e}^{-\psi b}, b \ge 0$$

where $\zeta$, $\uplambda ,$ and $\psi$ are the distribution parameters.

Runoff capture ratio ($RCR$), which can be defined as the percentage or ratio of the long-term average runoff volume captured and treated by drainage systems such as HFDs, is considered the best surrogate measure of the hydrologic performance of SuDS, such as biorentention systems^33^. Using design storms as an approach for designing drainage systems cannot straightforwardly calculate the $RCR$. Moreover, in certain situations, the design storm approach can lead to oversizing; in other cases, it can result in undersized drainage systems^34^. In any of these situations, the resulting impacts may include substantial environmental damages, such as flooding, erosion, disruption of local ecosystems, and increased operational and maintenance costs of urban infrastructures.

## Annual total overflow volume estimation

The $RCR$ of HFDs is evaluated by estimating the annual total overflow volume from HFDs. For a given design case, a random operation cycle is analyzed, beginning from the start of an interevent time (dry period) and ending at the end of the succeeding rainfall event. The annual total overflow is calculated as the product of the average overflow volume per operation cycle and the annual average number of operation cycles. As extensively discussed by Essien et al., interevent time (dry period) is statistically independent of succeeding rainfall event volume and duration^16^. At some locations, rainfall event duration and rainfall event volume were found to be weakly statistically dependent^16,34^. To simplify mathematical derivations, the statistical dependency between rainfall event volume and duration is usually neglected, and this simplification was found to be acceptable for many locations and various purposes^16,20,35,36^. Here, we adopted the same assumption in deriving our mathematical equations. Its acceptability will be verified by comparing it with continuous simulation results.

**Figure 2:**
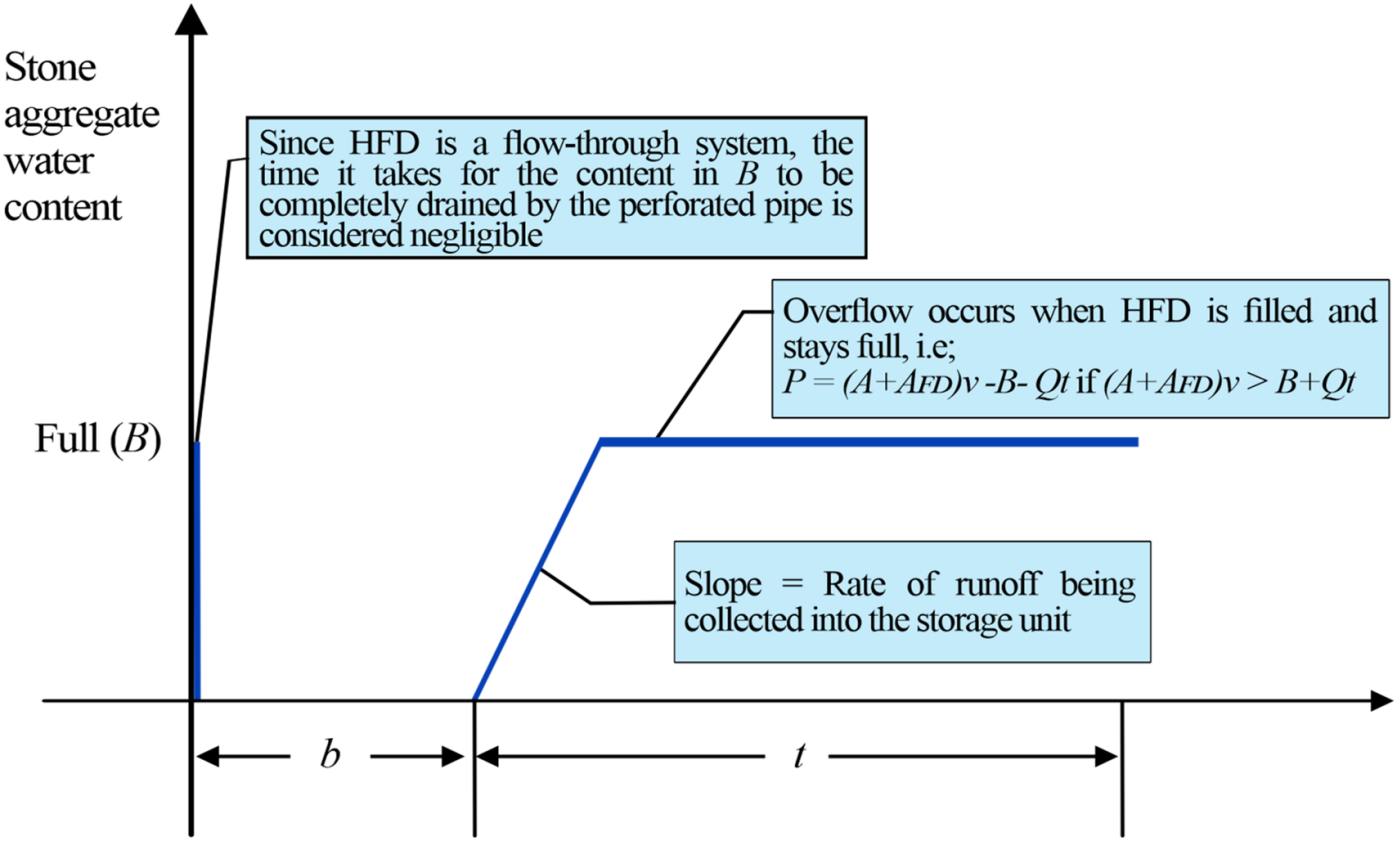
Demonstration of a completely emptied stone aggregate before the start of the analyzed rainfall event.

Based on the design principles of HFDs (HFDs design section), the overflow volume generated from a random operation cycle is influenced by the available void space in the stone aggregate at the start of the cycle. Since HFDs are largely considered as flow-through drainage systems as far as there is no extensive clogging of the stone aggregate, it can be assumed that the dry period preceding the rainfall event of the random operation cycle is always long enough to completely drain out runoff accumulated in the HFD before the start of the succeeding rainfall event. Therefore, the HFD is always completely empty at the beginning of the rainfall event in the random operation cycle. Figure 2 visually represents our assumptions and how the water content in an HFD changes within an operation cycle.

The total amount of the void space of the stone aggregate of an HFD is denoted as *B* (L), and the discharge rate from the underdrain perforated pipe under specific conditions of the HFD is denoted as $Q$(L/h). Here, $Q$ is treated as a constant for design purposes, similar to the assumption adopted by many jurisdictions and recommended by various authors for different SuDS designs^34,37–39^. In addition, for us to obtain a closed-form equation, we assumed that there is no side and bottom infiltration in the HFDs, which is typical for HFDs^6^.

As illustrated in Fig. 2, the stone aggregate of the HFD is completely emptied before the analysed rainfall event starts. During the analyzed rainfall event, the depth of the water in the HFD increases until the stone aggregate is completely full, and then overflow occurs if more runoff flows towards the HFD. Let the motorway surface area to be drained be $A$(m^2^), the surface area of the HFD be ${A}_{FD}$(m^2^), and the overflow volume be $p$ (L). The equation that expresses the condition resulting in an overflow is as follows: ($A$ + ${A}_{FD}$)$v$ $-B-Qt>0$; that is $v>(B+Qt)$/($A$ + ${A}_{FD}$). Here, we use ($A$ + ${A}_{FD}$)$v$ to represent the total volume of runoff flowing into an HFD due to a rainfall event with a volume $v$. We assumed that the surface of the motorway is completely impervious, therefore converting 100% of the rainfall event volume to surface runoff. This assumption implies that both evaporation and transpiration are negligible due to the sloped design of motorway surfaces, which aids the rapid flow of runoff to HFDs. The method for removing this simplifying assumption is discussed in the Methods section. Since $A$ and ${A}_{FD}$ are expressed in m^2^ and $v$ in mm, ($A$ + ${A}_{FD}$)$v$ has the unit of liters (L). Hence, there is no need for unit conversion in the expressions. However, if other unit systems are used for the variables, appropriate unit conversion will be required. The spill volume $p$ = ($A$ + ${A}_{FD}$)$v-B-Qt$ when ($A$ + ${A}_{FD}$)$v$ $-B-Qt>0$*,* is considered a random variable as its exact value depends on $v$ and $t$, which are assumed to be statistically independent random variables resulting from the analyzed random rainfall event. $p$ may be zero for some events, representing cases where there is no occurrence of overflow.

The expected value of the random variable $p$ can be derived based on the derived probability distribution theory. According to Benjamin and Cornell, this theory suggests that the probability distribution of a dependent random variable is intrinsically linked to and can be derived from those of the independent random variables^40^. This is achieved using the functional relationship that exists between the dependent and independent random variables. The expressions we previously used to describe the condition of overflow and the resulting overflow amount form a region of integration for determining the probability per rainfall event that the overflow volume equals or exceeds a specific value $p$. This resulted in the exceedance probability (i.e., the probability per operation cycle that overflow equals or exceeds a specific value $p$) expressed in Eq. (4), denoted as ${G}_{P}$($p$). The derivation of ${G}_{P}$($p$) is conducted by integrating the joint probability density function of $v$ and $t$. This results in Eq. (5).

4 $${G}_{P}\left(p\right)={\int }_{t=0}^{\infty }{\int }_{v = \left(p + B + Qt\right) / (A + {A}_{FD}) }^{\infty }\lambda {e}^{-\lambda t}{\zeta e}^{-\zeta \upsilon }d\upsilon dt$$

5 $${G}_{P}\left(p\right) = \lambda {e}^{-\zeta \left(\frac{p + B}{A +{ A}_{FD}}\right)}\frac{1}{\left[\lambda + \left(\frac{\zeta Q}{A+{A}_{FD}}\right)\right]}$$

In Eq. (4), $\lambda {e}^{-\lambda t}{\zeta e}^{-\zeta \upsilon }$ is the joint probability density function of $v$ and $t$. Since we assume that $v$ and $t$ are independent, their joint PDF is simply the product of their marginal PDFs. To calculate the probability that some overflow would occur per rainfall event, which is denoted as ${G}_{P}(0)$, $p$ is substituted with zero (i.e., $p$ = 0) in Eq. (5), which gives Eq. (6).

6 $${G}_{P}(0)=\lambda {e}^{-\zeta \left(\frac{B}{A + {A}_{FD}}\right)}\frac{1}{\left[\lambda + \left(\frac{\zeta Q}{A + {A}_{FD}}\right)\right]}$$

The probability of no occurrence of overflow per rainfall event is equal to (1 – ${G}_{P}(0)$), which is denoted as Prob($p$ = 0), and it is an impulse probability at $p$ = 0. The probability density function of the overflow volume per rainfall event is expressed as ${f}_{P}\left(p\right)$; and for $p$ > 0, it can be obtained from the negative derivative of ${G}_{P}\left(p\right)$ as expressed in Eq. (5). This derivation and its results are expressed in Eq. (7).

$${f}_{P}\left(p\right) = - \frac{d}{{d}_{p}}{G}_{P}\left(p\right) = \lambda {e}^{-\zeta \left(\frac{p + B}{A + {A}_{FD}}\right)}\frac{1}{\left[\lambda + \left(\frac{\zeta Q}{A + {A}_{FD}}\right)\right]}$$

7 $$=\lambda {e}^{-\zeta \left(\frac{p + B}{A + {A}_{FD}}\right)}\frac{1}{\left[\lambda + \left(\frac{\zeta Q}{A +{A}_{FD}}\right)\right]} \frac{\zeta }{(A+{A}_{FD})}=\frac{\zeta }{A+{A}_{FD}} {G}_{P}\left(p\right)$$

In addition, the complete probability distribution function of the overflow volume contains the impulse probability at $p$ = 0. Therefore, the expected value of the overflow volume per rainfall event can be determined using Eq. (8).

$$E\left(p\right)= 0 \times Prob\left(p=0\right)+ {\int }_{0}^{\infty }p{f}_{P}\left(p\right)dp$$

8 $$=\lambda {e}^{-\zeta \left(\frac{ B}{A + {A}_{FD}}\right)}\frac{1}{\left[\lambda + \left(\frac{\zeta Q}{A +{A}_{FD}}\right)\right]} \frac{(A + {A}_{FD})}{\zeta }=\frac{(A +{ A}_{FD})}{\zeta } {G}_{P}(0)$$

With Eq. (8), the annual total overflow volume denoted as ${p}_{AT}$ can be calculated by multiplying the expected value of the overflow volume per rainfall event by the average annual number of rainfall events when the HFD is in operation, denoted as *θ*, resulting in Eq. (9)

9 $${p}_{AT}=\theta \frac{(A +{ A}_{FD})}{\zeta } {G}_{P}(0)$$

Here ${G}_{P}(0)$ is expressed in Eq. (6).

## Evaluation of the runoff capture ratio (${\varvec{R}}{\varvec{C}}{\varvec{R}}$)

The total inflow volume (${v}_{T}$), expressed in mm of water over the surface of the HFD, is determined using Eq. (10). In Eq. (10), $r$ is a dimensionless design parameter evaluated as the ratio between the motorway surface area (i.e., the impervious surface area drained by HFD) and the surface area of the HFD. With Eq. (10) and the probability density function of $v$ expressed in Eq. (1), the expected value of ${v}_{T}$ per rainfall event can be calculated. This is expressed in Eq. (11), and the total annual volume of inflow onto the surface of the HFD can be determined using Eq. (12).

10 $${v}_{T}=(A +{ A}_{FD})\frac{v}{{ A}_{FD}} =(r + 1)v$$

11 $${E(v}_{T})={\int }_{0}^{\infty }(r+1)v\zeta {e}^{-\zeta v}dv= \frac{r + 1}{\zeta }$$

12 $${v}_{AT}={A}_{FD}\theta \left(\frac{r + 1}{\zeta }\right)= \frac{A}{r}\theta \left(\frac{r + 1}{\zeta }\right)$$

In Eq. (12), the annual total volume of inflow (${v}_{AT}$) is expressed in liters, and $\frac{A}{r}$ is equal to ${A}_{FD}$. Therefore, substituting $\frac{A}{r}$ in Eq. (6) for ${A}_{FD}$, ${G}_{P}(0)$ can also be expressed as in Eq. (13).

13 $${G}_{P}(0)=\lambda {e}^{-\zeta \left[\frac{B}{A\left(1 + \frac{1}{r}\right)}\right]}\frac{1}{\left[\lambda + \left(\frac{\zeta Q}{A\left(1 + \frac{1}{r}\right)}\right)\right]}$$

To estimate the annual volume of runoff captured by the HFD, denoted as ${v}_{FD}$ and expressed in Eq. 14, we subtracted the annual total overflow volume (${p}_{AT}$) from the annual total inflow volume (${v}_{AT}$).

$${v}_{FD}=\frac{A}{r}\theta \left(\frac{r + 1}{\zeta }\right) - \theta \frac{(A +{ A}_{FD})}{\zeta } {G}_{P}(0)$$

14 $$= \frac{A\theta \left(r+1\right)}{r\zeta } \left[1- {G}_{P}(0)\right]$$

Finally, the runoff capture ratio ($RCR$) can be expressed in Eq. (15).

15 $$RCR= \frac{{v}_{FD}}{{v}_{AT}}= 1- {G}_{P}(0)$$

To calculate the $$RCR$$ using Eq. (15), first, the area of the subcatchment drained by a given length of an HFD and the surface area of that length of the HFD must be known as well as the void space ($$B)$$ in the length of the HFD. In addition, the rainfall distribution parameters of the location of interest must also be known, and then the probability per rainfall event that some overflow occurs $$\left[{G}_{P}(0)\right]$$ is evaluated using Eq. (6) or Eq. (13) (if the area ratio ($$r$$) is explicitly used). In addition to the other advantages of using this APM, the analytical equations presented in this paper make $$RCR$$ more tractable, allowing for easier examination and understanding of the inter-relationships between the variables involved. For example, $$B$$ can be solved from Eq. (13) if $${G}_{P}(0)$$ is given or specified, the result is reported in Eq. (16).

16 $$B= \frac{- \left[{{\ln}}\left(\frac{{G}_{P}(0)\left[\lambda + \left(\frac{\zeta Q}{A+ {A}_{FD}}\right)\right]}{\lambda }\right)\right] \left(A+ {A}_{FD}\right)}{\zeta }$$

Equation (16) will be very useful during the preliminary phase of HFD designs as it directly determines the required storage capacity of HFDs for achieving a specified level of $RCR$. This is because Eq. (15) shows that ${G}_{P}(0)=1-RCR$, therefore given $RCR$ is related to given ${G}_{P}(0)$, substitute a given or specified ${G}_{P}(0)$ into Eq. (16), the required *B* is easily determined. For example, if the specified $RCR$ of a planned HFD is 0.98, then ${G}_{P}(0)$ used in Eq. (16) can be obtained by subtracting this pre-determined *RCR* from 1 (i.e., 1–0.98).

In addition to ensuring that the void spaces of HFDs are large enough for capturing high enough percentages of runoff from their contributing areas, their effective void space should also be large enough to help filter out pollutants such as microplastics, heavy metal, oil and grease, etc., from runoff^12^, thus protecting aquatic ecosystems. Moreover, as discussed by Rowland and Ellis, in terms of infrastructure resilience and waste management issues, with the increasing impacts of climate change, ensuring resilient drainage systems, such as HFDs, becomes paramount^12^; trenches with the right void space can also reduce the strain on HFDs, prolonging their lifespan, useability, and reducing unnecessary drain fouling due to the potential impact of climate change.

## Validation of the APM

To validate the APM, we evaluated the $$RCR$$ of 6 design examples for a location in each of the four nations of the UK. The 55-year hourly rainfall data we used to verify the exponentiality of the selected stations in the four nations of the UK were used as the input rainfall data to SWMM models (more information about the verification of the exponentiality of the rainfall distribution of the locations in the UK is in the Data collection section)^16^. The results from our model (the APM) were compared to those estimated by the U.S. EPA's Storm Water Management Model (SWMM). Details of the comparison results are discussed in the subsequent section (the Discussion section). Although our APM provides a straightforward method to evaluate the hydrologic performance of an HFD, SWMM does not. To estimate the $$RCR$$ based on SWMM simulation results, Eq. (17) is used, and the relative difference ($$RD)$$ in percentage between both models is evaluated using Eq. (18).

17 $${RCR}_{SWMM}= \frac{{V}_{TI}- {V}_{SO}}{{V}_{TI}}$$

where ${V}_{TI}$ is the total inflow volume into the HFD in mm, i.e., the total runoff generated over the motorway surface and the rainfall depth onto the surface of the HFD calculated by SWMM using the 55-year hourly rainfall data, and ${V}_{SO}$ is the total surface outflow volume in mm from the HFD, i.e., the total overflow volume from the HFD. ${V}_{SO}$ is simply the unfiltered runoff volume from the HFD.

18 $$RD= \frac{|{RCR}_{APM}- {RCR}_{SWMM}|}{{RCR}_{SWMM}} \times 100$$

## Discussion

**Figure 3:**
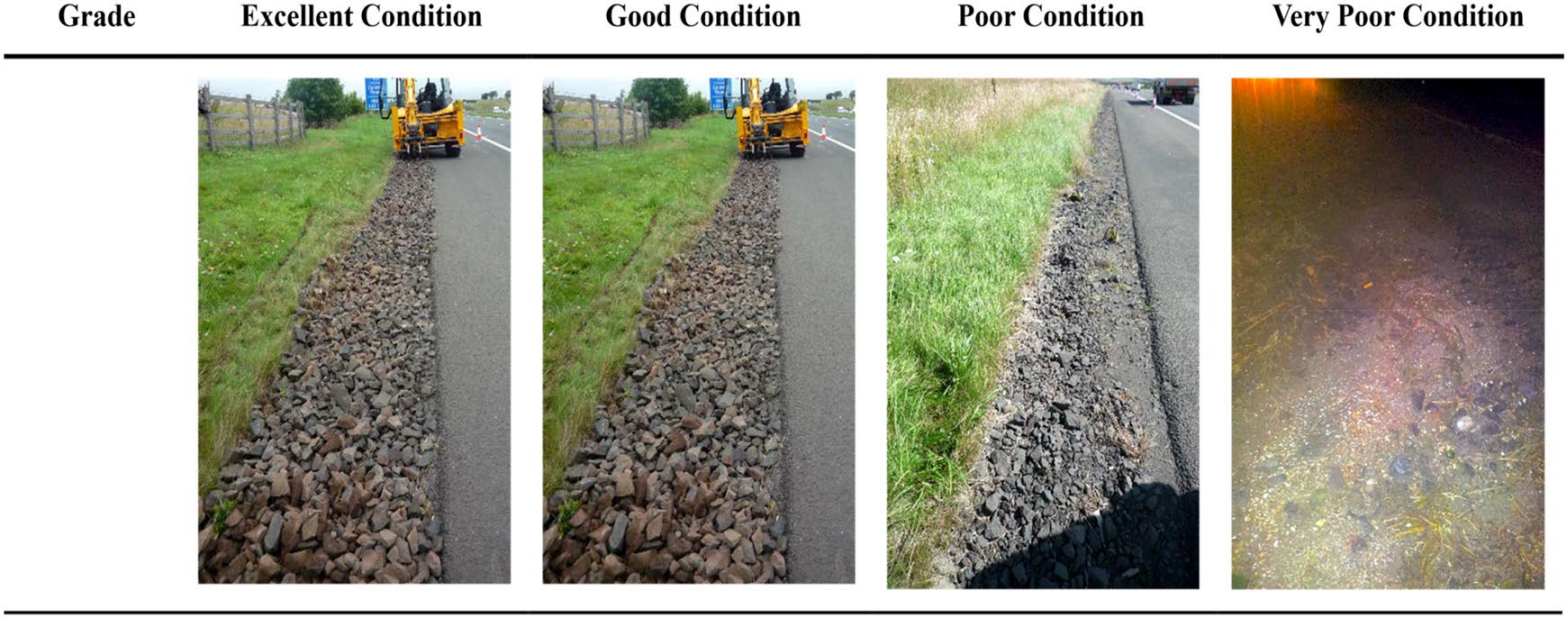
Visual representation of HFD conditions observed in the UK^15^. The excellent condition was as a newly constructed HFD, and the good condition had superficial defects but no obvious fouling^15^. The poor condition had moderate to high levels of fouling (i.e., part of the stone aggregate at the surface may not be visible at some angle), and the very poor condition possessed extreme foulants (i.e., the stone aggregate at the surface was not visible)^15^.

In the previous sections, we have demonstrated how we developed all the equations for our APM. Here, we present the results of the design examples we used to validate our APM. The validation examples encompass 6 design cases in each of the four nations of the UK. For the first 4 validation cases, HFDs were classified into four band conditions based on their level of fouling. The four levels of fouling were defined following the visual assessment of the HFD conditions conducted in the UK^15^. Figure 3 provides a graphical demonstration of the four levels of fouling.

Supplementary Table 2 shows the summary linking the void ratio and normalised permeability determined through lab experiments to the visually assessed four band conditions of HFD and other input parameter values used in SWMM simulations. Based on the relationship between the normalised permeability and the drain coefficient of the excellent-condition HFD, we calculated the percentage reduction of the drain coefficient for the good-, poor-, and very poor-condition HFDs. A study by Nelsen et al. on drain trenches established that their sampled contaminants in the drain trenches were found more at the bottom of the drain trenches^41^. Recognizing that contaminants or obstructions, called foulants, accumulate more over time at the HFD's underdrain area than in other parts, we added an additional 2% to the calculated reduction used to estimate the resulting drain coefficient ($C$) to account for these real-world variables and ensured a safety margin for conservativeness (Supplementary Table 2). Validation examples 5 and 6 are design cases where the width of the HFDs were reduced from the "1 m × 1 m" UK industry standard and a hypothetical design case, respectively.

Validation examples 1–4 (i.e., excellent, good, poor, and very poor design cases) are 2 to 6-lane motorways drained by 1 m × 1 m HFDs. This is because the typical number of lanes in UK motorways is between two to six. Examples are the ones in M25 close to Heathrow Airport^42^, where 66% of the total length is estimated to be treated by HFDs^43^. The subcatchment (i.e., the motorway surface area) drained by the HFDs are the impervious pavements with a length of 50 m and a width corresponding to the number of lanes. For example, a 3-lane motorway has a width of 10.95 m, i.e., 3 multiplied by 3.65 m (the average width of a single motorway lane in the UK)^44–47^. The length of 50 m was used for the validation examples as HFDs run parallel with the surface of motorways, and manholes are typically provided in the UK at a maximum interval of 90 m^48^. Validation examples 1–4 (i.e., VE 1–4) assessed the hydrologic performance of the four band conditions of HFDs with input parameter values shown in Table 1.

**Table 1:** SWMM simulation input parameters for validation examples 1–6.

Parameter	Excellent condition (VE1)	Good condition (VE2)	Poor condition (VE3)	Very poor condition (VE4)	Validation example 5	Validation example 6
Motorway surface area (i.e., $A$) (ha)	0.0365–0.1095	0.0365–0.1095	0.0365–0.1095	0.0365–0.1095	0.1095	0.1825
*S* (%)	2.5	2.5	2.5	2.5	2.5	2.5
$n$	0.011	0.011	0.011	0.011	0.011	0.011
HFD's area (i.e., $${A}_{FD})$$ (ha)	0.005	0.005	0.005	0.005	0.0023–0.0043	0.0023
HFD's stone aggregate thickness (mm)	925	925	925	925	925	375
HFD's void ratio (i.e., *e*)	0.7	0.5	0.3	0.1	0.7	0.1
Drain coefficient ($C$)	60	30	3	0.6	71–133	1
${n}_{FD}$	0.5	0.5	0.5	0.5	0.5	0.5

*S* is the cross slope of the motorway surface area, $n$ is Manning's roughness coefficient of the motorway surface, and ${n}_{FD}$ is the drain exponent of the HFD. APM does not require *S* and $n$. In both models, evaporation during rainfall was assumed to be negligible. For the 55-year hourly rainfall records used as the rainfall input data (excluding the winter months, as urban stormwater management systems are typically designed based on rainfall statistics of non-winter months)^16,27^, the calibration of SWMM simulations indicated that using a time step of 30 min or shorter rendered the model notably less sensitive to time step length.

**Figure 4:**
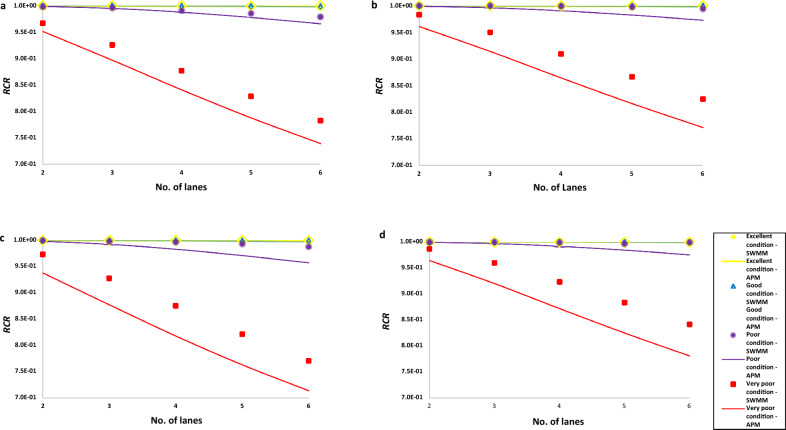
SWMM vs APM runoff capture ratios ($RCR$ s) for the four band conditions' design cases in the selected stations in England (**a**), Scotland (**b**), Wales (**c**), and Northern Ireland (**d**). Shown are the marker and line plots representing the $RCR$ estimated by SWMM and APM, respectively. The four band conditions of HFDs are described in four different colours. The $RCR$ for the top two band conditions were all approaching 1; hence, the overlapping of their plots. The legends by the side of figure (**d**) represent figure (**a**)–(**c**).

Figure 4 compares the $RCR$ calculated by APM to the ones estimated by SWMM for the four band conditions of HFD. The figure shows that the results calculated by the APM are all in close agreement with those estimated by SWMM. Using Eq. (18), the relative differences ($RDs$) of the $RCR$ estimated by the two models are shown in Table 2 for the four band conditions. In the four design cases, the maximum relative difference of 7.37% was observed from the very poor-condition HFDs for the location in Wales. It was observed that the estimated $RCR$ decreases as the band condition of the HFDs drops. This is expected since poor-condition HFDs have lower drain coefficients. One important observation is that SWMM overestimated the $RCR$ in all the design cases, except for the $RCR$ estimated for the poor condition design case on the 2-lane motorway in England's location, which was slightly overestimated by the APM (i.e., 0.9986 and 0.9990 from SWMM and APM, respectively). The $RCR$ values determined by the APM tend to be conservative. This is partly because in determining the spill volume per rainfall event, APM assumes that rainfall falling onto the motorway surface immediately reaches the HFD surface while SWMM models in detail the flows of runoff over the motorway surface with the input of two parameters (i.e., *S* and $n$). As the flows over the motorway surface take some time, the possible spill volume from the HFD would be slightly reduced, and SWMM-simulated $RCR$ would be slightly higher. In addition, it's essential to recognize that while the APM provides a simplified approach for calculating the $RCR$, certain nuances and dynamic behaviors of stormwater from the motorway and filtration through HFDs during rainfall events might not be fully captured, which also lead to observed underestimations. This may perhaps balance out some simplifying assumptions used for the development of APM, specifically the use of a constant discharge rate based on the highest level of saturated stone aggregate. Nevertheless, the conservative nature of the APM makes it more suitable for the preliminary design or quick assessment of the hydrologic performance of HFDs.

**Table 2:** Relative difference between SWMM and APM determined $RCR$ s for the four band conditions' design cases in each location of the four nations of the UK.

No. of lanes	Excellent condition				Good condition				Poor condition				Very poor condition			
	England	Scotland	Wales	Northern Ireland	England	Scotland	Wales	Northern Ireland	England	Scotland	Wales	Northern Ireland	England	Scotland	Wales	Northern Ireland
2	0.00	0.00	0.00	0.00	0.00	0.00	0.00	0.00	0.04	0.07	0.19	0.06	1.61	2.24	3.61	2.24
3	0.00	0.00	0.01	0.00	0.02	0.01	0.03	0.01	0.08	0.37	0.57	0.31	3.11	3.78	5.42	4.15
4	0.01	0.01	0.02	0.01	0.02	0.04	0.08	0.03	0.34	0.85	1.37	0.72	4.10	4.94	6.53	5.53
5	0.01	0.02	0.04	0.02	0.08	0.09	0.15	0.07	0.78	1.49	2.26	1.24	4.96	5.78	7.07	6.61
6	0.03	0.05	0.07	0.04	0.11	0.15	0.24	0.14	1.38	2.15	3.15	2.42	5.55	6.48	7.37	7.24

The relative difference is expressed in %.

**Figure 5:**
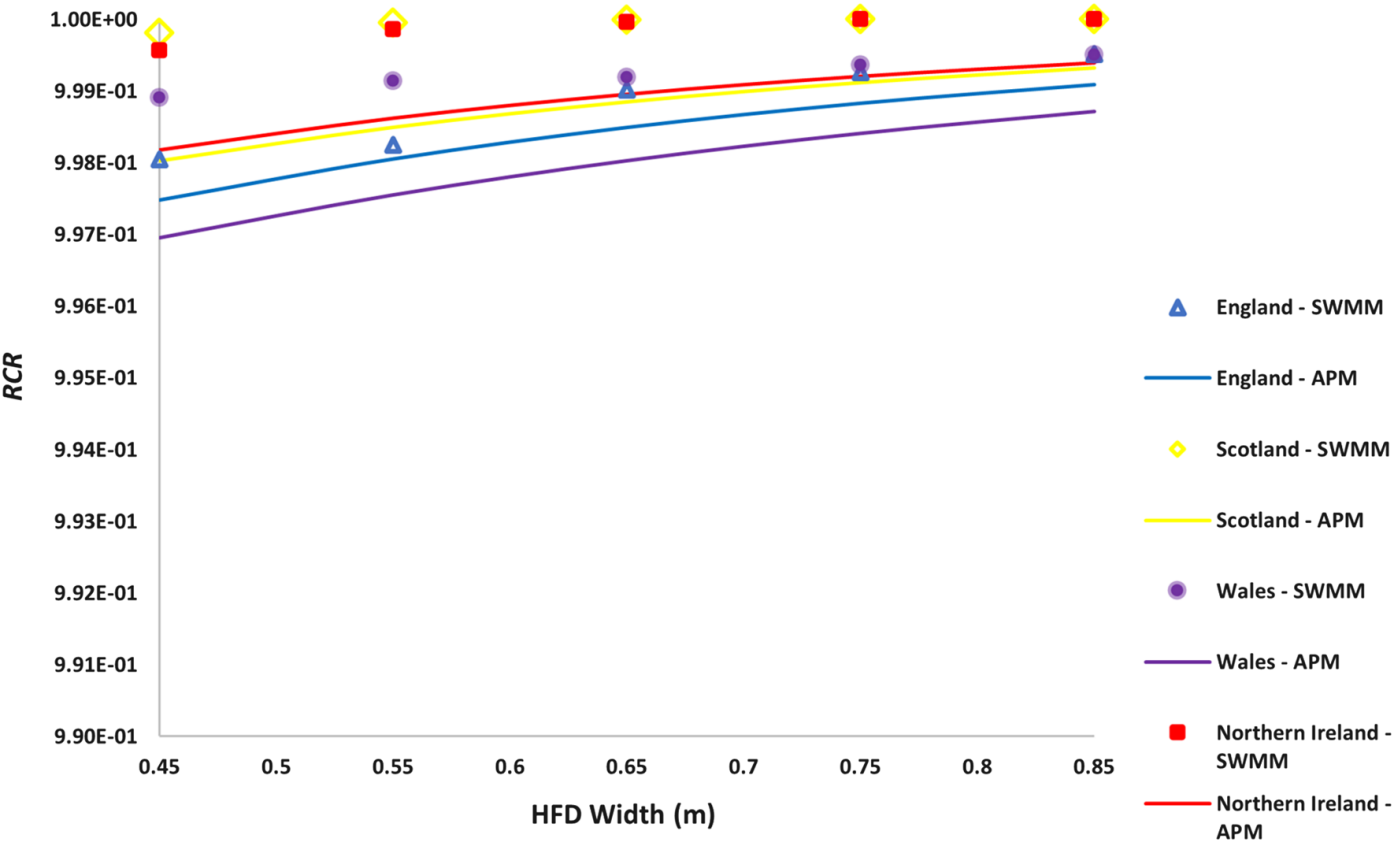
Validation example 5 SWMM vs APM determined $RCR$ s for each location in the UK. Shown here are the marker and line plots in different colours representing the $RCR$ estimated by SWMM and APM, respectively.

Validation example 5 was conducted to evaluate the impact of the reduction of the width of HFD on its hydrologic performance. For this validation example, instead of the standard 1 m × 1 m HFD design model typically used in the UK, simulations for 0.45 m × 1 m to 0.85 m × 1 m design cases were conducted. Figure 5 shows the $RCR$ for the locations in each of the UK nations. Both models indicate in Fig. 5 that if the width of HFDs is reduced to 0.45 m, a minimum $RCR$ of 0.997 can still be achieved. Therefore, we propose that the UK highway construction industry adopt HFDs with dimensions of 0.45 m × 1 m. This recommendation aligns with the sizing criteria stipulated by Highway England (i.e., the minimum width of HFD must be 300 mm plus the underdrain pipe diameter), as illustrated in Fig. 1. The suggested width reduction can significantly reduce the resources needed to construct HFDs, thereby reducing cost while still maintaining the high hydrologic performance. In addition, it can reduce the land and carbon footprint, which is one of the key environmental issues associated with drainage systems^49,50^. When the design storm approach is used to size HFDs for flood controls as detailed by Highway England (Supplementary Section 1), the longitudinal slope, diameter and roughness of the different segments of the perforated pipe should all be taken into consideration to verify that the recommended reduced width of the HFD is still large enough to avoid the flooding of the motorway.

Validation example 6 is a hypothetical design case aimed at assessing how robust the APM is at estimating the hydrologic performance for HFDs with $RCR$ less than 0.50. For validation example 6, it was found that the *RCR*s are 0.32 vs 0.30 for England's location, 0.37 vs 0.34 for Scotland's location, 0.31 vs 0.29 for Wales's location, and 0.37 vs 0.35 for Northern Ireland's location as estimated by SWMM and APM, respectively. Although from the hydrologic performance results of the four band conditions discussed earlier, it may be unlikely to have HFDs under this condition, to confirm the robustness of the APM, it was necessary to test it out and observe that the relative difference of the *RCR*s between both models for the four locations in the UK are all < 8.5%.

### Recommendation

In some cases, typically motorways with cuttings, HFDs are used as combined drainage systems (i.e., for both surface and subsurface drainage)^27^. This is because providing separate drainage systems for the surface and subsurface water is often difficult. The current version of SWMM and APM does not directly consider subsurface drainage for HFDs. Although considering the subsurface drainage by HFDs may be complex due to the complexity of the groundwater flows to the HFDS, resulting from seasonal variations and heterogenous soil properties, it may have a significant impact and needs to be factored into both models in the future.

## Conclusion

Our work presents the first analytical probabilistic model (APM) for designing and estimating the hydrologic performance of highway filter drains (HFDs). HFD is widely used in many countries for stormwater runoff management. We derived the analytical equations for calculating the runoff capture ratio ($RCR$) of HFDs based on the probabilistic models of rainfall characteristics and a simplified representation of the hydrological processes that take place in the operation of HFDs. Coded into a spreadsheet, APM can be a straightforward and easy-to-use tool. Hence, we highly recommend that academic researchers, industry, and government agencies take advantage of this simple tool, especially at the preliminary design and performance evaluation stages of HFDs, or to estimate the hydrologic performance of existing HFDs under different operating conditions.

The results from the validation examples demonstrated that the relative differences ($RDs$) of the runoff capture ratios for all the simulated cases in the four nations of the UK were all less than 8.5%. We also suggest that to save cost through resource reduction needed to construct HFDs as well as reduction of land and carbon footprints, HFD width can be reduced from the UK industry standard width of 1 m to 0.45 m as both widths provide approximately the same hydrologic performance. Based on both models, 0.997 is the lowest runoff capture ratio reached when the width of 0.45 m was used.

Finally, by using a simplified approach, the APM can be adapted for different locations worldwide. Different design dimensions (i.e., HFD surface area and depth) may be tried, and their corresponding $B$, $r$, and $Q$ values estimated and substituted into Eq. (15). The traditional method of using a design storm-based or continuous simulation approach doesn't directly provide an estimate of runoff capture ratio, while the APM can, and it does so much more effectively.

## Methods

We next describe more with sample data about the verification of the exponential distributions of the observed rainfall characteristics recorded in selected stations in the four nations of the UK, the simplifying assumptions adopted in the derivation of the APM that are not adopted in SWMM, and the relationship between APM and SWMM input parameters.

### Data collection

As previously discussed in the Derivation of the analytical equations for assessing the hydrologic performance of HFDs section, taking a statistical approach with the aim to obtain closed-form equations that allow for the direct calculation of $RCR$ of HFDs requires the verification of probabilistic models of the rainfall event characteristics of the specific locations of interest. A study by Essien et al. has extensively discussed the procedures for doing this for the four nations of the UK in a way that has never been done before^16^. Table 3 shows the probabilistic model distribution parameters for the study areas used in this paper^16^.

**Table 3:** Rainfall distribution parameters for selected stations in the four nations of the UK^16^.

Country name	Weather station name	Distribution parameters				
		ζ (mm^−1^)	λ (hr^−1^)	*Ψ* (hr^−1^)	*θ*	$\tilde{V}_{{{\text{aat}}}}$(mm)
England	Heathrow	0.158	0.110	0.012	71	450
Scotland	Turnhouse and Gogarbank	0.165	0.095	0.015	82	500
Wales	Rhoose and St Athan	0.134	0.094	0.017	90	666
Northern Ireland	Aldergrove	0.171	0.095	0.019	103	603

The rainfall distribution parameters were estimated using the method of moments. They are the reciprocals for the mean values of the rainfall event characteristics $(v, t,\, {\text{and}}\, b)$, and $\tilde{V}_{{{\text{aat}}}}$ represents the average annual total rainfall volume.

### Simplifying assumptions adopted by APM

In this paper, we have mentioned several times about the simplifying assumptions we adopted after understanding the design and operating principles of HFDs in order to derive the analytical equations. The following is a summary of all the simplifying assumptions:

- The rainfall event characteristics $(v\, {\text{and}}\, t)$ are random variables that are exponentially distributed, and they are statistically independent of each other.
- The runoff from the impervious pavement immediately flows towards the HFD, assuming no shoulder or verge in between the motorway lanes' surface area and the HFD as this intermediate area may be either paved or unpaved.
- The spill volume for the analyzed random rainfall event is calculated based on the assumption that the rainfall intensity remains constant throughout the event. In other words, uniform intensities are assumed for each rainfall event.
- The HFD trench's stone aggregate void space is analyzed in detail using probabilistic methods, specifically focusing on the operation cycle that follows the preceding rainfall event. At the end of the dry period following the preceding rainfall event, the void space of the stone aggregate is assumed to be completely empty.
- As long as there is water in the stone aggregate of the HFD, the rate of discharge through the perforated pipe remains constant. Infiltration into the native soils is negligible, and the impact of gradual clogging of the pipes, which would gradually reduce the discharge rate, is considered to be insignificant.

### Modeling HFD using SWMM

SWMM is a free, open-source software used worldwide for planning, analysis, and design-related stormwater runoff modelling with a special module for simulating SuDS^51^. This module is called the LID Control Editor^51^. With this module, HFD can be modeled via a three-layer tab, of which two are used for the HFD simulation. These are storage (thickness and void ratio) and drain (flow coefficient and flow exponent)^51^.

### Establishing the relationship between APM and SWMM input parameters

Here, the void space ($B$) in the stone aggregate of an HFD is related to its void ratio ($e$), defined as the ratio between the volume occupied by the empty spaces and the volume occupied by the solids. The relationship between the APM and SWMM parameters with respect to the total void space in the stone aggregate of an HFD ($B$) is established in Eq. (19).

19 $$B= \frac{e{A}_{FD}h}{1+e}$$

where $B$ is in L if ${A}_{FD}$ is in m^2^ and $h$ is in mm. Using Eq. (19), we may determine the value of $B$ required to reach a specific $RCR$ using Eq. (16) first; afterwards, we can compute the required depth ($h$) of the stone aggregate using Eq. (20).

20 $$h= \frac{\left(1+e\right)B}{e{A}_{FD}}$$

According to the latest SWMM manual, “if the drain consists of slotted pipes where the slots act as orifices, then the drain exponent would be 0.5 and the drain coefficient would be 60,000 times the ratio of total slot area to LID area. For example, drain pipe with five 1/4" diameter holes per foot spaced 50 feet apart would have an area ratio of 0.000035 and a drain coefficient of 2”^51^. So, as stated in the Supplementary Section 1, jurisdictions within the UK require a minimum of 1000 mm^2^ holes per 1000 mm length of perforated pipe used in HFDs. Using this minimum requirement, we can calculate the drain's discharge rate based on the properties of the perforated pipe using Eq. (21).

21 $$\it {\text{Q}}= {{\text{A}}}_{{\text{FD}}}{\text{C}}{{{\text{h}}}_{{\text{s}}}}^{{{\text{n}}}_{{\text{FD}}}}$$

where $C$ and ${n}_{FD}$ are the drain coefficient and exponent, respectively, ${h}_{{\text{s}}}$ is the height of the saturated stone aggregate above the perforated pipe's impermeable bedding. As mentioned in the Discussion section, the maximum possible ${h}_{{\text{s}}}$ was used for the APM in this study. The typical value for ${n}_{FD}$ is 0.5^51^, which, therefore, significantly reduces the impact of the saturation level of the storage aggregate on the discharge rate.

More importantly, SWMM can model depression storage of the surface area to be drained by filter drains. To include the effect of depression storages in the APM, distribution parameter $\zeta$, estimated using the average rainfall event volume ($\overline{v }$) of a location of interest may be modified. The modified $\zeta$, denoted as ${\zeta }_{m}$, can be calculated using Eq. (22).

22 $${\zeta }_{m}= \frac{1}{\overline{v }-{S}_{d} }$$

where ${S}_{d}$ is the depression storage in mm of the surface area to be drained by filter drains. $(\overline{v }-{S}_{d})$ ensures that, before generating runoff that will flow towards the HFD, the rainfall amount equivalent to the depression storage of the surface of the motorway is removed from every individual rainfall event.

### Supplementary Information

Supplementary Information.

## Data Availability

The hourly rainfall data recorded at selected stations in the four nations of the UK was emailed by the UK meteorological office (Met Office) to the authors upon request. The data can be requested by contacting the Met Office via the following email: enquiries@metoffice.gov.uk or available via the following link: https://www.metoffice.gov.uk/research/library-and-archive. The secondary data referenced in the paper have been published and are currently open access. The static Figures were processed with Microsoft 365 version and CorelDraw X8, and the numeric data for all the plots are provided in this paper as source data or contact A.E. (essiena@mcmaster.ca).

## Code availability

The continuous modeling was conducted using SWMM 5.2 (https://www.epa.gov/water-research/storm-water-management-model-swmm), which is open-source, public software and is free for use worldwide.
