# Author Correction: Design and hydrologic performance estimation of highway filter drains using a novel analytical probabilistic model

**DOI:** 10.1038/s41598-024-56781-0

**Published:** 2024-03-18

**Authors:** Aniekan E. Essien, Yiping Guo, Mohamed Khafagy, Sarah E. Dickson-Anderson

**Affiliations:** 1 Department of Civil Engineering, McMaster University, Hamilton, ON L8S 4L7 Canada; 2 Irrigation and Hydraulics Department, Faculty of Engineering, Cairo University, Giza, Egypt

Correction to: *Scientific Reports* 10.1038/s41598-024-52760-7, published online 29 January 2024

The original version of this Article omitted an affiliation for Mohamed Khafagy. The correct affiliations are listed below.

Department of Civil Engineering, McMaster University, Hamilton, Ontario L8S4L7, Canada

Irrigation and Hydraulics Department, Faculty of Engineering, Cairo University, Giza, Egypt

The original Article has been corrected.

