# Supplementary material for: Design and hydrologic performance estimation of highway filter drains using a novel analytical probabilistic model
Source: Sci Rep. 2024 Jan 29;14:2350. doi: 10.1038/s41598-024-52760-7 (PMC10824712; doi:10.1038/s41598-024-52760-7)
Supplement: Supplementary file 1 — Supplementary Information. [file 41598_2024_52760_MOESM1_ESM.docx]

**Article Supplementary Information**

| **Design and hydrologic performance estimation of highway filter drains using a novel analytical probabilistic model** |
| --- |

Aniekan E. Essien^a^, Yiping Guo^a📧^, Mohamed Khafagy^a^, & Sarah E. Dickson‐Anderson ^a📧^

a Department of Civil Engineering, McMaster University, Hamilton, Ontario, Canada, L8S 4L7.

📧e-mail: [guoy@mcmaster.ca](mailto:guoy@mcmaster.ca); [sdickso@mcmaster.ca](mailto:sdickso@mcmaster.ca)

**S1 The UK standard design criteria for highway filter drains (HFDs)**

- Storm event requirement of 1 in 1 year with HFD to drain and accommodate flows in bore without surcharge and 1 in 5 years with HFD to drain and accommodate flows without causing chamber surcharge levels to exceed the formation level. In simple terms, HFDs should be designed to manage rainfall events with a 1 in 1 year return period without overflow from the HFD stone aggregate or 1 in 5 years without flooding on the motorway surface.
- The typical internal diameter of HFD conveyor pipes that are laid with a longitudinal gradient greater than 0.25% ranges from 100 to 375 mm, and the backfill on the pipe is directly related to the pipe's outside diameter, thereby resulting in the following formula: pipe outside diameter (X) + 300 mm = HFD width (Y).
- The two major types of carrier pipe used for HFDs are steel and plastic (although plastic pipes have been known to be the UK's favourite drainage pipes since the 1940s). Both types of pipes must be perforated with a minimum of 1000 mm^2^ holes per 1000 mm length of pipe, and the pipe stiffness due to perforations shall not be reduced by more than 5%. The diameter of circular perforations must range between 3 – 10 mm, and rectangular perforations must have holes ≤ 4 mm in length and ≥ 0.6 mm in width.
- The granular materials (Type A, B, and C) used as infill for HFDs, which are conventionally but not restrictively exposed at the surface of the system, must be porous in nature. Although for the piping system, the bedding material on which the conveyor pipe is laid must be less permeable than the backfill. Therefore, type A material (0 – 20 mm) is the standard underfill and type B material (20 – 40 mm) is the standard backfill's aggregate. Supplementary Table 1 shows the standard specification for particle size distributions (PSD) as stated by the National Highways, UK.
- HFDs shall be a minimum of 600 mm below the sub-base of the pavement, and the height of the carrier pipe bedding shall not be less than 75 mm.

**Table S1 Aggregates grading requirement for HFDs in the UK**^1^

| **British standard sieve size** | **Percentage by mass passing** | |
| --- | --- | --- |
|  | **Type A material** | **Type B material** |
| 80 | – | 100 |
| 63 | – | 98 - 100 |
| 40 | 100 | 80 - 99 |
| 20 | 80 - 99 | 0 - 20 |
| 10 | 50 - 90 | 0 - 5 |
| 4 | 30 - 75 | – |
| 2 | 15 - 60 | – |
| 0.500 | 0 - 35 | – |
| 0.125 | 0 - 4 | – |
| 0.063 | 0 - 3 | – |

**Table S2 HFD band conditions and their corresponding void ratios, normalised permeabilities, and drain coefficients (**$\boldsymbol{C}$**)**

| **HFD Band condition**^2^ | **Void ratio**^2^ | **Normalized permeability**^2^ | **% reduction + safety margin of 2%** | **Estimated** $\boldsymbol{C}$ |
| --- | --- | --- | --- | --- |
| Excellent | 0.7 | 0.67 | - | 60 |
| Good | 0.5 | 0.35 | 50 | 30 |
| Poor | 0.3 | 0.05 | 95 | 3 |
| Very Poor | 0.1 | 0.02 | 99 | 0.6 |

Here, HFD in excellent condition, which has a normalised permeability of 0.67, has a drain coefficient ($C$) of 60. To calculate the $C$ for HFD in good condition, we set the value of 0.67 as the baseline, representing 100%. The revised value, post-reduction, is 0.35. To quantify the reduction, we subtract the new value from the original, finding an absolute decrease of 0.32 (i.e., 0.67 - 0.35). To express this decrease as a percentage of the baseline value, we calculate the ratio of the absolute reduction (0.32) to the baseline value (0.67) and multiply it by 100, yielding a percentage reduction of approximately 47.76%. This calculation indicates that the reduction from 0.67 to 0.35 equates to nearly halving the original value, with the new value representing just over half of the base value. We added the 2% safety margin to the 47.76%, which approximately gives 50%. Consequently, 50% of estimated excellent condition HFD's $C$ result in the value of 30 for the $C$ of the good condition HFD. The same steps are followed to calculate the $C$ for the poor and very poor condition HFDs.

**References**

1. National Highways. *MANUAL OF CONTRACT DOCUMENTS FOR HIGHWAY WORKS VOLUME 1 SPECIFICATION FOR HIGHWAY WORKS*. https://www.standardsforhighways.co.uk/ha/standards/mchw/vol1/pdfs/MCHW Vol 1 Series 500 web PDF.pdf (2020).

2. Stylianides, T., Frost, M. W., Fleming, P. R., Mageean, M. & Huetson, A. A Condition Assessment Approach for Highway Filter Drains Using Ground Penetrating Radar. in *Procedia Engineering* (eds. Hloch, S. & Krolczyk, G.) vol. 143 1226–1235 (Elsevier, 2016).
